# Draft Genome Sequences of Two Clinical Actinobacillus pleuropneumoniae Serotype 19 Strains from Pigs in Switzerland

**DOI:** 10.1128/MRA.00588-21

**Published:** 2021-08-26

**Authors:** Sophie Peterhans, Marc J. A. Stevens, Nicole Cernela, Xaver Sidler, Roger Stephan, Simone Scherrer

**Affiliations:** a Section of Veterinary Bacteriology, Vetsuisse Faculty, University of Zurich, Zurich, Switzerland; b Institute for Food Safety and Hygiene, Vetsuisse Faculty, University of Zurich, Zurich, Switzerland; c Department of Farm Animals, Division of Swine Medicine, Vetsuisse Faculty, University of Zurich, Zurich, Switzerland; University of Arizona

## Abstract

Actinobacillus pleuropneumoniae serotype 19 is a very recently described new serotype with a novel type II capsule synthesis locus. Here, we report the draft genome sequences of two Actinobacillus pleuropneumoniae serotype 19 strains with a serogroup 3/6/8/12/15 O-antigen locus that were isolated in 2018 and 2021 from two different pig farms in Switzerland.

## ANNOUNCEMENT

Actinobacillus pleuropneumoniae is a contagious lung pathogen in pigs and the etiological agent of porcine pleuropneumonia, which is responsible for global economic losses (1). Based on the capsule synthesis (*cps*) genes, A. pleuropneumoniae can be classified into 19 serotypes with different importance in pathogenicity (2, 3). Based on a novel type II capsule synthesis locus, serotype 19 has recently been described in Denmark and Canada (3). Two A. pleuropneumoniae serotype 19 strains, 18-1342 and G1-9626, were isolated by the Section of Veterinary Bacteriology at the University of Zurich in 2018 and 2021, respectively, from lungs of diseased pigs from two farms in Switzerland. The lung specimens were cultured on chocolate agar at 37°C for 48 h in 5% CO_2_. The isolated strains were subcultured on chocolate agar, the serotype was determined by multiplex PCR as described (2, 3), and the *apx* profile was identified (4).

Several colonies of a pure agar culture were picked for genomic DNA isolation using the DNA blood and tissue kit (Qiagen, Hombrechtikon, Switzerland). DNA libraries were prepared with the Nextera DNA Flex sample preparation kit (Illumina, San Diego, CA, USA), and the resulting libraries were sequenced on an Illumina MiniSeq sequencer. Up to 16 sequencing libraries were analyzed in one lane, and demultiplexing was performed using the GenerateFASTQ option in MiniSeq Local Run Manager v2.4.1.

The sequencing outputs were 890,468 and 783,131 paired-end reads of 150 bp for strain 18-1342 and strain G1-9626, respectively. The genome coverage was approximately 110-fold for both strains. The Illumina reads passed a quality check performed with FastQC v0.11.7 (Babraham Bioinformatics, Cambridge, UK) and were assembled with the SPAdes v3.13.1-based software Shovill v1.1.0 (5, 6), using default settings. The assembly was filtered, retaining contigs of >500 bp, and annotated using the NCBI Prokaryotic Genome Annotation Pipeline (PGAP) (7).

The draft genome of strain 18-1342 has a size of 2,187,672 bp divided over 50 contigs, with an *N*_50_ value of 123,287 bp and an *L*_50_ value of 6. Strain G1-9626 consists of 2,188,052 bp in 52 contigs, with an *N*_50_ value of 123,364 bp and an *L*_50_ value of 6. The GC content of both genomes is 41.2%. Strain 18-1342 is predicted by the NCBI PGAP to contain 2,103 genes, and strain G1-9626 is predicted to contain 2,108 genes.

The *cps* loci identified in strains 18-1342 and G1-9626 were found to be 100% identical at the nucleotide level. In comparison to the previously published sequence of Danish strain 7213384-1 (GenBank accession no. MT468887) (3), one mismatch in *cps19C* was identified.

In an analysis of the lipopolysaccharide O-antigen sequences, 18-1342 differs from the Danish strain (GenBank accession no. MT468889) in only one nucleotide, whereas G1-9626 additionally contains a deletion of 37 bp within the locus of a putative acyltransferase family protein. BLASTn analysis of the O-antigen loci of 18-1342 and G1-9626 revealed that they share over 99% nucleotide identity with those of serovars 3, 8, 12, and 15 and over 97% with that of serovar 6, thus possessing a serogroup 3/6/8/12/15 O-antigen locus. As described (3), both strains are predicted to produce ApxII, as they contain *apxIIAC* and *apxIBD* and harbor *apxIVA*. These genomes of two A. pleuropneumoniae isolates demonstrate the presence of serotype 19 in Switzerland for at least 3 years.

### Data availability.

This whole-genome shotgun project has been deposited in DDBJ/ENA/GenBank under the accession no. JAHHZT000000000 (strain 18-1342) and JAHHZU000000000 (strain G1-9626). The versions described in this paper are SAMN19375790 and SAMN19375791. The raw sequencing reads for strain 18-1342 (accession no. SRX11015211) and strain G1-9626 (accession no. SRX11015212) have been deposited in the SRA.
